# Comparison of short-term costs and 3-year complications in geriatric femoral neck fractures: hemiarthroplasty versus total hip arthroplasty

**DOI:** 10.1186/s12891-026-09598-z

**Published:** 2026-04-21

**Authors:** Bike Li, Tao Cui, Ming Ling, Cong Chen, Fuzhi Yang, Yongqian Fan

**Affiliations:** 1 Department of Orthopedics, Huadong Hospital, Fudan University, 221 Yan’an West Road, Jing’an District, Shanghai, 200040 China; 2 Department of Thoracic Surgery, Shanghai Key Laboratory of Clinical Geriatric Medicine, Huadong Hospital, Fudan University, Shanghai, 200040 China

**Keywords:** Hemiarthroplasty, Total hip arthroplasty, Inverse probability of treatment weighting, Age-adjusted Charlson Comorbidity Index, Costs, Complications

## Abstract

**Background:**

Hemiarthroplasty (HA) and total hip arthroplasty (THA) are the primary surgical options for geriatric femoral neck fractures (FNF). Our study balanced heterogeneity in baseline patient conditions and included long-term follow-up data, compared short-term costs and reoperation risks within the 3-year period, aimed to provide evidence for personalized surgical selection.

**Methods:**

This study enrolled 878 patients aged ≥ 60 years with first-time FNF treated at the Orthopedics Department of a large Grade-Three Class-A Hospital in China (2013.03–2021.12), categorized into HA and THA groups. Collected demographic characteristics and relevant clinical data, and Inverse Probability of Treatment Weighting (IPTW) was applied to adjust for baseline heterogeneity. Primary outcomes encompassed prosthesis-related complications, secondary fractures included contralateral fracture and osteoporotic vertebral compression fracture (OVCF), and short-term costs. Subgroup analyses stratified by age (60–75 vs. ≥ 75 years) and age-adjusted Charlson Comorbidity Index (aCCI < 5 vs. ≥ 5) were conducted to identify high-risk populations.

**Results:**

The THA group demonstrated significantly higher risks of periprosthetic fracture (OR = 2.43, 95% CI: 1.09–5.41; *P =* 0.030) and dislocation (OR = 4.27, 95% CI: 1.44–12.66; *P* = 0.009) compared to the HA, but had a reduced risk of deep vein thrombosis (DVT) (OR = 0.26, 95% CI: 0.11–0.63; *P =* 0.003). In high-risk subgroups (age ≥ 75 and aCCI ≥ 5), we observed dramatically increased risks of periprosthetic fracture and dislocation, but there were no differences in low-risk subgroups except for DVT. Short-term cost analysis revealed that the THA incurred higher hospitalization expenses [$13,038 ($9,047–$14,795) vs. $9,043 ($8,012–$10,178)].

**Conclusion:**

THA carries an elevated risk of mechanical complications within 3 years, especially among patients aged ≥ 75 years or with an aCCI ≥ 5. For these high-risk populations, HA should be prioritized to mitigate catastrophic risks. THA incurs higher short-term medical costs, but it may benefit low-risk patients through long-term functional improvement.

**Supplementary Information:**

The online version contains supplementary material available at 10.1186/s12891-026-09598-z.

## Introduction

The accelerated global aging has led to a continuous rise in the incidence of FNF among the elderly [1]. In Asia, China’s age-standardized incidence rate in 2019 was estimated at 117.8 per 100,000 population (95% CI, 83.8-161.6) [2]. The average treatment expense for hip fractures reaches $7,443, nearly equivalent to the annual income of many Chinese households [3]. Additional costs include long-term rehabilitation expenses and indirect losses from work disabilities [4]. These fractures have evolved into a global public health crisis, urgently requiring evidence-based individualized treatment strategies.

Currently, HA and THA are the primary surgical approaches for geriatric FNF, but selection strategies remain controversial. HA offers shorter operative time, less intraoperative blood loss, and lower costs, making it suitable for elderly patients or those with multiple comorbidities [4]. However, it carries risks of acetabular wear and revision. Moon and Kang found that the average linear and volumetric degenerative changes of acetabular cartilage at 2 years after HA were 0.23 ± 0.107 mm/year and 114 ± 47.2 mm3/year [5], respectively; Dayama and Olorunfemi found a 5.3% revision rate in patients under 75 of HA [6]. THA demonstrates significantly higher postoperative Harris scores and lower long-term revision rates [7], but involves higher surgical costs, dislocation risks (4.7% vs. 2.4%), and elevated periprosthetic fracture risks [8]. Recent studies further reveal that THA reduces reoperation risk compared to HA (OR = 0.83), while dislocation risk remains significantly higher within 4 years post-THA (OR = 1.31) [9]. Although THA incurs higher initial costs ($11,421 vs. $7,799), its long-term revision expenses ($17,130 vs. $22,006) and indirect costs (home care, rehabilitation) are lower [10, 11]. Despite international guidelines recommending THA for patients with higher activity demands, existing evidence has two major limitations. First, insufficient adjustment for baseline heterogeneity—most studies inadequately control for age, comorbidities, and bone density differences, leading to biased risk estimates [12]; second, short-term follow-up limitations—90% of randomized controlled trials only evaluate ≤ 2-year outcomes, neglecting long-term risks like secondary fractures (15% 5-year incidence) and prosthesis wear (32% 20-year failure rate) [13].

This study based on data from 878 elderly FNF patients, employed IPTW to balance age, comorbidities, aCCI, and osteoporosis status, eliminating confounding bias (SMD < 0.1) [14]. It systematically evaluated reoperation rates, secondary fractures, and short-term costs, with stratification by age (60–75 vs. ≥75 years) and comorbidities (aCCI < 5 vs. ≥ 5), identifying high-risk THA populations. Our findings are expected to provide evidence-based support for personalized treatment of geriatric FNF and offer data-driven insights for efficient healthcare resource allocation.

## Methods

### Participants and data collection

**Fig. 1:**
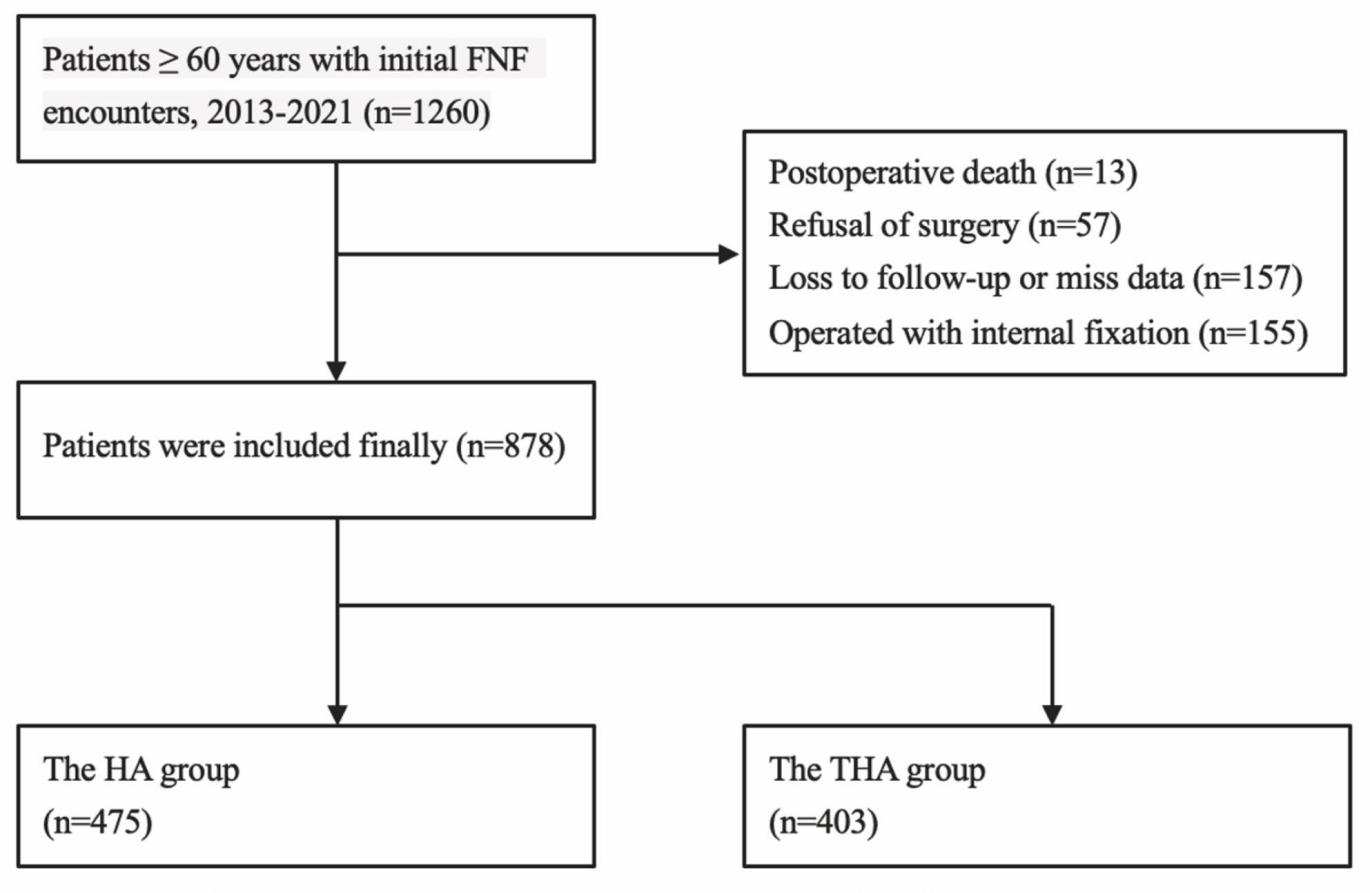
Based on the exclusion criteria, a final total of 475 patients were included in the HA group and 403 patients in the THA group

This study was a single-center retrospective cohort study that included 1,260 elderly patients (≥ 60 years) with FNF treated at the Orthopedics Department of a large Grade-Three Class-A Hospital in China from March 2013 to December 2021. Patients were excluded based on the following criteria: postoperative death (*n* = 13), refusal of surgery (*n* = 57), loss to follow-up or missing data (*n* = 157), and internal fixation treatment (*n* = 155). Ultimately, 878 patients were enrolled and divided into HA (*n* = 475) and THA (*n* = 403) groups (Fig. 1). The choice between HA and THA was determined by the attending surgeon in consultation with the patient and family, based on a comprehensive assessment of the patient’s chronological and physiological age, pre‑fracture activity level, cognitive status, intraoperative bone quality, presence of symptomatic hip osteoarthritis, and life expectancy. This was a clinical, non‑randomized decision.

Two independent orthopedic surgeons collected the following information through electronic medical records and telephone follow-ups: (1) Demographic and Clinical Characteristics: Age, Sex, Body Mass Index (BMI), Interval between fracture and operation, Comorbidities (hypertension, diabetes, osteoporosis, etc.), calculate the aCCI. (2) Outcome Measures: The primary outcomes were complications leading to reoperation within 3 years (including periprosthetic fracture, dislocation, periprosthetic infection, DVT, and wound nonunion) and direct medical hospitalization costs. Secondary outcomes included secondary fractures (contralateral hip and OVCF), length of stay, operative parameters (duration, blood loss) and postoperative pain assessed by the VAS. Verbal informed consent was obtained from all participants after full disclosure of the research objectives and procedures. The study protocol was approved by the Institutional Ethics Committee (Approval No. 2022K062) and conducted in accordance with the ethical principles of the Declaration of Helsinki.

### Operation and perioperative management

All surgeries were performed by the same experienced surgical team. The HA group received biological femoral stem and a bipolar ceramic head, while the THA group received biological femoral stem, a bipolar ceramic head, a metal acetabular cup, and a ceramic liner, with all products supplied by Zimmer (*Zimmer Biomet*,* Warsaw*,* Indiana*,* USA*). Intraoperative C-arm fluoroscopy confirmed satisfactory component positioning and limb length, with good joint stability.​​ Preoperative low molecular weight heparin for DVT prophylaxis; intravenous infusion of antibiotics (cefuroxime) 30 min before skin incision. Postoperative management follows the principle of “early mobilization and delayed weight-bearing” [15, 16]. Rehabilitation training, including muscle strength training and gait adjustment, was designed by rehabilitation specialists. Ultrasonographic screening for DVT was performed within 48 h postoperatively. Anti-osteoporosis therapy was administered in accordance with clinical guidelines. Regular outpatient follow-ups assess prosthesis stability and functional recovery [17].

### Data processing and analysis

Missing values of continuous variables were addressed using multiple imputation (*mice* package in R), and missing values of categorical variables were assigned to the “Unknown” category [18]. After re-examination of the electronic medical record system, corrected or retained outliers.​​ IPTW was applied to adjust for baseline heterogeneity, propensity scores (PS) were calculated via logistic regression [19], with covariates including age, sex, BMI, interval between fracture and operation, comorbidities (hypertension and 14 others), and aCCI stratification (< 5: low-medium risk; ≥ 5: high risk), with SMD < 0.1 indicating balance. Weighted chi-square, Yates-corrected chi-square, or Fisher’s exact tests compared categorical outcomes; weighted Mann-Whitney U tests analyzed continuous variables. Subgroup analyses stratified by age (60–75 vs. ≥ 75 years) and aCCI (< 5 vs. ≥ 5) assessed THA-related risks and describe it with forest maps [20]. Analyses were performed in R 4.4.3 (*Matchlt* package for IPTW, *survey* package for weighted data). Two-sided tests with α = 0.05 were applied, and *P* < 0.05 was considered statistically significant.

## Results

### Balancing effect of heterogeneity in baseline

Before IPTW, the THA group had a significantly lower mean age compared to the HA group (72.39 ± 7.08 years vs. 82.76 ± 6.39 years, SMD = 1.542). The prevalence rates of listed comorbidities (e.g., hypertension, diabetes) were all lower in the THA group (all SMD > 0.1). The aCCI index distribution showed the HA group had a higher proportion in the aCCI ≥ 5 subgroup (64.63%), while the THA group dominated in the aCCI < 5 subgroup (73.20%) (all SMD > 0.1). After IPTW adjustment, all covariates achieved SMD < 0.1, indicating effective baseline balance by the model (Table 1).

**Table 1:** Balancing baseline heterogeneity in elderly FNF: SMD before and after IPTW

Variable	HA	THA	SMD before IPTW	SMD after IPTW
Patients, n (%)	475(54.10)	403(45.90)		
Mean age (SD)	82.76(6.39)	72.39(7.08)	1.542	0.046
Sex, n (%)			0.117	0.009
Men	119(25.05)	122(30.27)		
Women	356(74.95)	281(69.73)		
BMI	21.00(3.45)	22.28(3.25)	0.379	0.057
Interval	6.72(5.30)	5.91(5.93)	0.145	0.075
Comorbidity, n (%)				
Hypertension	229(48.21)	139(34.49)	0.281	0.007
Diabetes	103(21.68)	58(14.39)	0.191	0.010
Cerebrovascular disease	88(18.53)	41(10.17)	0.240	0.040
Coronary artery disease	51(10.74)	13(3.23)	0.298	0.008
Parkinson	18(3.79)	5(1.24)	0.163	0.011
Dementia	15(3.16)	2(0.50)	0.200	0.033
Atrial fibrillation	17(3.58)	3(0.74)	0.196	0.026
Tumor	34(7.16)	18(4.47)	0.115	0.015
Pneumonia	20(4.21)	11(2.73)	0.081	0.004
Liver disease	6(1.26)	9(2.23)	0.074	0.021
Chronic renal disease	7(1.47)	4(0.99)	0.044	0.004
aCCI, n (%)				
< 5	168(35.37)	295(73.20)	0.082	0.037
⩾5	307(64.63)	108(26.80)	0.082	0.037
OP	457(96.2)	331(82.1)	0.465	0.006

*Interval* Interval between fracture and operation, *HA* Hemiarthroplasty, *THA* Total hip arthroplasty, *aCCI* Age-adjusted Charlson Comorbidity Index, *OP* Osteoporosis, *SMD* Standardized Mean Difference, *IPTW* Inverse Probability of Treatment Weighting

### Reoperation within 3 years

After IPTW, there was no statistically significant difference in the 3-year reoperation rate between the THA and HA groups (OR = 1.39, 95% CI: 0.90–2.16; *P* = 0.139). However, the THA group showed significantly higher risks of periprosthetic fracture (OR = 2.43, 95% CI: 1.09–5.41; *P* = 0.030) and dislocation (OR = 4.27, 95% CI: 1.44–12.66; *P* = 0.009), while demonstrating a reduced risk of DVT (OR = 0.26, 95% CI: 0.11–0.63; *P* = 0.003). No significant differences were observed in periprosthetic infections (OR = 3.77, 95% CI: 0.42–34.09; *P* = 0.235) or wound nonunion rates (OR = 0.93, 95% CI: 0.41–2.15; *P* = 0.888). Secondary fractures, including contralateral fractures (OR = 1.06, 95% CI: 0.55–2.06; *P* = 0.843) and OVCF (OR = 1.52, 95% CI: 0.69–3.34; *P* = 0.302), also showed no statistical differences between groups (Table 2).

**Table 2:** Weighted comparisons of reoperation and secondary fractures before and after IPTW

Variable	HA *n* (%)	THA *n* (%)	Before IPTW		After IPTW	
			OR (95% CI)	*P*	OR (95% CI)	*P*
Patients	475(54.10)	403(45.90)				
Reoperation within 3 years	52(10.95)	65(16.13)	1.56(1.06, 2.31)	0.024	1.39(0.90, 2.16)	0.139
Reasons for reoperation						
Periprosthetic fracture	12(2.53)	22(5.46)	2.23(1.09, 4.56)	0.025	2.43(1.09, 5.41)	0.030
Dislocation	5(1.05)	19(4.73)	4.65(1.72, 12.57)	0.001	4.27(1.44, 12.66)	0.009
Periprosthetic infection†	1(0.21)	4(0.99)	4.75(0.53, 42.69)	0.278	3.77(0.42, 34.09)	0.235
DVT	22(4.63)	8(1.99)	0.42(0.18, 0.95)	0.031	0.26(0.11, 0.63)	0.003
Wound nonunion	12(2.53)	12(2.98)	1.18(0.53, 2.67)	0.683	0.93(0.41, 2.15)	0.888
Secondary fractures						
Contralateral fracture	25(5.26)	19(4.71)	0.89(0.48, 1.64)	0.710	1.06(0.55, 2.06)	0.843
OVCF	15(3.16)	17(4.22)	1.35(0.67, 2.74)	0.403	1.52(0.69, 3.34)	0.302

†Yates-corrected chi-square

*OR* Odds ratio, *P* P-value

### Short-term costs

All short-term costs showed non-normal distributions and were described using medians (interquartile ranges). The THA group had significantly higher expenses [$13,038 ($9,047–$14,795) vs. $9,043 ($8,012–$10,178), *P* < 0.001], operation duration [120 (100–130) min vs. 70 (60–90) min, *P* < 0.001), and intraoperative blood loss [250 (200–300) mL vs. 100 (100–193) mL, *P* < 0.001] compared to the HA group. However, no statistically significant differences were observed in length of hospitalization stay [17.7 (14.9–22.8) days vs. 17.0 (13.6–21.8) days, *P* = 0.403] or postoperative VAS scores [3 (2–5) vs. 3 (2–5), *P* = 0.093] (Table 3).

**Table 3:** Analysis of short- term costs after IPTW

Variable	HA median(Q1-Q3)	THA median(Q1-Q3)	*P*-value	
			before IPTW	After IPTW
Length of stay (day)	17.0 (13.6–21.8)	17.7 (14.9–22.8)	0.031	0.403
Expenses ($)	9,043 (8,012 − 10,178)	13,038 (9,047 − 14,795)	< 0.001	< 0.001
Operative duration (min)	70.0 (60.0–90.0)	120.0 (100.0-130.0)	< 0.001	< 0.001
Blood loss (ml)	100.0 (100.0-193)	250.0 (200.0-300.0)	< 0.001	< 0.001
Vas	3.0 (2.0–5.0)	3.0 (2.0–5.0)	0.036	0.093

Q1-Q3: interquartile range

### Subgroup analysis

**Fig. 2:**
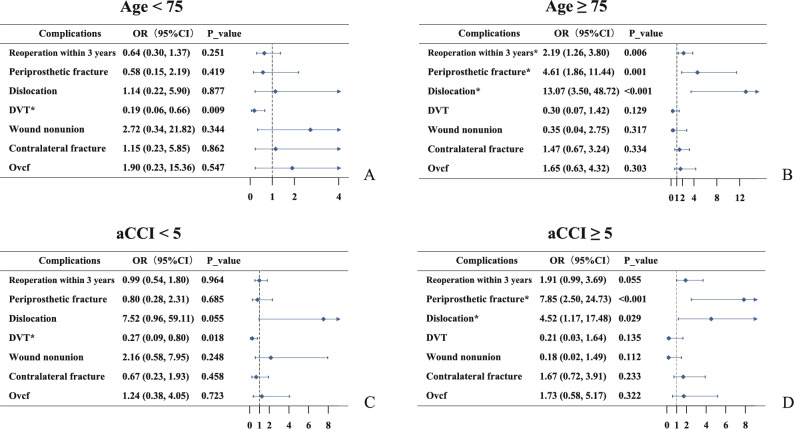
Subgroup analysis stratified by age and aCCI

When stratified by age, the THA group aged ≥ 75 years had significantly increased risks of periprosthetic fractures (OR = 4.61, 95% CI: 1.86–11.44; *P* = 0.001) and dislocation (OR = 13.07, 95% CI: 3.50-48.72; *P* < 0.001). The TAH group aged 60–75 years showed reduced DVT risk (OR = 0.19, 95% CI:0.06–0.66; *P* = 0.009). When stratified by aCCI, in the high-risk group (aCCI ≥ 5), the THA group exhibited significantly higher risks of periprosthetic fractures (OR = 7.85, 95% CI: 2.50-24.73; *P* < 0.001) and dislocation (OR = 4.52, 95% CI: 1.17–17.48; *P* = 0.029). In the low-risk group (aCCI < 5), only reduced DVT risk was observed (OR = 0.27, 95% CI: 0.09–0.80; *P* = 0.018), with no significant differences in other complications (Fig. 2).

## Discussion

This study compared the cost-effectiveness differences between HA and THA in elderly patients with FNF through a large-sample retrospective cohort. We acknowledge that the choice between THA and HA in clinical practice is a complex, individualized decision based on a multitude of factors, as reflected in our non-randomized study design. The goal of this analysis was not to challenge the rationale of this clinical decision-making but to evaluate, after robust statistical adjustment for measurable baseline heterogeneity, the comparative outcomes associated with the two treatment pathways as they are implemented in a real-world setting. The innovation of our research is reflected twofold: first, the use of the IPTW method effectively corrected baseline heterogeneity long neglected in previous studies [14, 21, 22]; second, it quantifies the sharp increase in mechanical complication risks of THA in the advanced-age (≥ 75 years) and high comorbidity burden (aCCI ≥ 5) subgroups. By comparing eight-year single-center data, HA group patients were older and had heavier comorbidity burdens (e.g., higher proportion of aCCI ≥ 5), which aligns with clinical practice favoring less traumatic HA procedures for advanced-age or high-risk patients [23].

This study found that THA significantly increases prosthesis-related mechanical complications and short-term economic burdens. The risk of periprosthetic fracture and dislocation at 3 years postoperatively in the THA group was 2.43 times and 4.27 times higher than in the HA group, respectively. These results align with the retrospective study of 10,268 patients by Adam I. Edelstein et al., which showed significantly elevated 12-month dislocation risk in THA for FNF (2.9% in THA vs. 1.9% in HA; *P* = 0.001) [24]. This phenomenon relates closely to THA’s prosthesis design characteristics. THA requires removal of acetabular cartilage and implantation of acetabular cups, compared with HA’s dual-mobility design, THA’s joint center more closely approximates anatomical positioning, theoretically reducing impingement risks [25–28]. However, this advantage may be offset by age-related bone quality deterioration - osteoporotic patients’ inadequate acetabular cup fixation strength could lead to stress concentration at the micro-motion interface [29], while THA’s extensive soft tissue dissection may exacerbate capsular stability damage [30]. Subgroup analysis revealed no difference in periprosthetic fracture/dislocation risks among patients < 75 years or aCCI < 5, but dramatic risk surges emerged in age ≥ 75 or aCCI ≥ 5 subgroups: periprosthetic fracture (OR = 4.61, *P* = 0.001 and OR = 7.85, *P* < 0.001) and dislocation (OR = 13.07, *P* < 0.001 and OR = 4.52, *P* = 0.029). These findings corroborate the conclusions of Podmore and Hutchings that advanced age or comorbidities amplify THA’s mechanical complication risks [31]. Importantly, the significant baseline differences before adjustment, reflecting the clinical selection of HA for frailer patients, and the persistent elevation of mechanical risks after statistical balancing, jointly underscore that the procedural demands of THA pose a disproportionately higher risk for the very elderly and comorbid patients typically considered for HA. Additionally, we identified that mechanical complication rates were not low in either the HA or THA groups. This phenomenon may be attributable to the study’s single-center design within a developing country, where patients exhibited high postoperative labor demands and suboptimal adherence to structured rehabilitation and anti-osteoporosis regimens. However, these hypotheses warrant further validation through multicenter studies with larger cohorts.

We observed a significantly reduced DVT risk in the THA group (OR = 0.26), which contradicts conventional assumptions that THA’s longer operative time and greater trauma should theoretically increase venous stasis risks [32, 33]. Despite IPTW adjustment for measurable covariates, the HA group’s inherent profile—older age and greater comorbidity burden—is associated with higher blood viscosity, delayed mobilization, and reduced activity levels, all predisposing to stasis [34]. This interpretation is supported by our subgroup analysis, where the significant DVT risk reduction associated with THA was confined to the younger (< 75 years) and lower-risk (aCCI < 5) subgroups, and was absent in the older and higher-risk subgroups. Another critical consideration is rehabilitation protocols and postoperative anticoagulation regimens, though no differences existed between groups in our treatment protocols [35].

The short-term cost analysis revealed that the median hospitalization cost for THA was 44.18% higher than HA, aligning with international multicenter studies’ cost difference trends [36]. However, it should be noted that this study did not incorporate longer-term follow-up data, while existing evidence suggests THA may reverse cost-effectiveness through reduced revision rates over 10-year follow-ups, with a THA 10-year revision rate of 13% [37], while a 10-year follow-up of 114 HA patients showed 38.6% eventually converted to THA [38], yet our 3-year observation window likely missed such events. Therefore, for low-risk subgroups (aCCI < 5 and Age < 75), despite higher short-term costs, THA remains a reasonable choice—particularly for patients with higher activity demands, as THA’s long-term functional advantages might translate into indirect cost savings [39]. These potential long-term functional benefits warrant validation in subsequent studies.

### Limitations

This study has several limitations. First, its single-center, retrospective design inherently carries risks of selection bias. Although we employed IPTW to balance observable covariates, residual confounding may persist due to unrecorded factors influencing surgical selection, such as detailed cognitive status, pre-fracture activity level, fall risk, and surgeon preference. Second, our analysis lacked patient-reported functional outcomes (e.g., Harris Hip Score), which are crucial for a comprehensive assessment of treatment effectiveness. Third, the 3-year follow-up, while providing mid-term insights, is insufficient to evaluate long-term outcomes critical to this debate, including implant survivorship, late mechanical failure, or the potential long-term functional and cost advantages of THA. Fourth, specific clinical practices at our institution should be considered: (1) data on adherence to postoperative anti-osteoporosis therapy was not systematically captured, which may influence secondary fracture rates; (2) the routine use of biological femoral stems in all patients, including those over 75, represents a practice variation that may have impacted periprosthetic fracture rates and affects the generalizability of our results.

## Conclusions

Elderly patients undergoing THA have a higher incidence of prosthesis-related complications within 3 years post-operation, with advanced age or multiple comorbidities amplifying this risk. Those high-risk populations should opt for HA, while low-risk groups choosing THA require full disclosure of trade-offs between short-term cost increases and potential long-term functional improvements. Clinically, an aCCI-stratified decision-making pathway should be established, alongside enhanced mechanical complication monitoring during the first 3 years post-THA.

## Supplementary Information

Supplementary Material 1.

## Data Availability

All data analyzed during this study are included in the supplementary information files.

## Abbreviations

HA: Hemiarthroplasty
THA: Total hip arthroplasty
FNF: Femoral neck fracture
IPTW: Inverse Probability of Treatment Weighting
OVCF: Osteoporotic vertebral compression fracture
aCCI: Age-adjusted Charlson Comorbidity Index
DVT: Deep vein thrombosis
